# New Developments in Nutraceuticals and Functional Food Products: Microorganisms as Alternative Sources of Nutritive and Beneficial Components

**DOI:** 10.3390/foods12122321

**Published:** 2023-06-09

**Authors:** Carolina Chiellini, Duccio Cavalieri, Morena Gabriele

**Affiliations:** 1 Institute of Agricultural Biology and Biotechnology, Italian National Research Council, Via Moruzzi 1, 56124 Pisa, Italy; carolina.chiellini@ibba.cnr.it; 2 Department of Biology, University of Florence, Via Madonna del Piano 6, 50019 Sesto Fiorentino, Italy; duccio.cavalieri@unifi.it

Microorganisms have long been essential to human life, playing significant roles in food and beverage production, health and disease, and the environment. Nowadays, microorganisms represent an innovative biotechnological option and a source of bioactive and functional molecules for making new ingredients, novel foods, and functional formulations [1]. Products and components derived from microorganisms might have beneficial effects on human and animal health and can be successfully used in the food and feed industry, as well as in the nutraceutical, cosmetic, and pharmaceutical fields. Accordingly, microbes and microbial processes represent alternative sources of nutritive and beneficial/functional components and an alternative strategy to obtain products with implemented nutritional and health features. From microalgae to probiotics and beyond, the use of microorganisms in food production and nutrition has opened up new developments in research and innovation.

This Special Issue focused on new developments in nutraceuticals and functional food products exploiting microbial processes and microorganisms themselves as alternative sources of nutritive and functional properties. It gathers papers exploring the use of microalgae as alternative food sources, due to their potential as functional feedstocks for foods, feeds, supplements, and nutraceutical formulations, as well as an eco-friendly strategy to reduce the environmental impact of some traditional food production. Microalgae can be grown in small areas and do not require large amounts of water, making them a sustainable food source. In addition, microalgae are rich in nutrients, making them an excellent source of protein and other essential nutrients.

In this context, Chiellini et al. [2] analyzed and compared 11 microalgal strains from freshwater environments for their nutraceutical properties, focusing on the phytochemical profile and in vitro antioxidant activities. The results helped identify four strains as potential candidates for simultaneous massive growth and bioactive compound production and showed that biochemical parameters and antioxidant activities varied based on the solvents and applied treatment rather than microalgae strains. These findings may have implications for the development of sustainable and healthy food products. According to Macaluso et al. [3], the same microorganisms can also play a role in addressing environmental issues, e.g., reducing the polluting potential derived from different traditional food processing, such as olive oil mill wastewater (OMWW), which is a serious pollutant in the Mediterranean countries due to its high content of tannins, polyphenols, polyalcohols, pectins, and lipids. Researchers demonstrated that microalgae could be a low-cost and eco-friendly solution for OMWW treatment, and the application of microalgae could be developed as a full-scale approach within companies to obtain fortified microalgal biomass for nutraceutical fields.

The use of selected microorganisms and mathematical modeling are two additional topics that are currently gaining attraction in the field of food science. The Special Issue follows with a series of articles exploring the application of these concepts to various food products. Among these, the study reported by Bianchi et al. [4] focused on the effects of early inoculation of *Lactobacillus plantarum*, a homolactic acid bacteria for hexoses, on Sangiovese wine quality and longevity compared with traditional inoculation at the end of the alcoholic fermentation of *Oenococcus oeni*, a heterolactic bacterial strain for hexoses. The research found that early inoculation with *L. plantarum* resulted in higher phenolic content in two out of the three analyzed wines, even maintained during wine aging in wood. The results suggest that early inoculation with *L. plantarum* could be a beneficial winemaking technique for Sangiovese grape varieties.

In contrast, Zeng et al. [5] investigated the mechanism by which Lactobacillus, isolated from Chinese fermented foods, promotes the secretion of 5-hydroxytryptophan (5-HTP), a substance thought to improve depression. The study highlighted that *L. pentosus* LPQ1 displayed the highest 5-HTP secretion-promoting effect, which was dependent on a mixture of compounds secreted by the bacteria (termed SLPQ1) able to alter the TNF and oxidative phosphorylation signaling pathways. A similar 5-HTP promoting effect was also observed using the SLPQ1 ultrafiltration fraction >10 kDa. The findings add information on the mechanism by which *L. pentosus* LPQ1 promotes 5-HTP production in some cell lines in vitro.

Additionally, Cela et al. [6] evaluated, in a soilless system with sub-optimal phosphorus, the effect of *Funneliformis mosseae*, an arbuscular mycorrhizal fungus (AMF), on the growth and nutritional quality of lettuce plants (*Lactuca sativa* L. cv. *Salinas*) compared with that on non-inoculated controls. The results showed that mycorrhizal inoculation improved lettuce plants’ growth and nutritional quality, even at sub-optimal phosphorus concentrations. Moreover, AMF plants showed larger phytochemical content, as well as higher antioxidant capacity values than control plants grown at optimal P nutrition levels. Moreover, the amount of leaf gas exchange was higher in AMF-inoculated plants, and the nitrogen, phosphorus, and magnesium leaf levels were increased compared with that in non-inoculated plants grown at the same P level. These findings suggest that *F. mosseae* can improve the nutritional quality and can stimulate the growth of lettuce plants even at sub-optimal P concentrations.

The last two articles of this series used statistical analysis and mathematical modeling to optimize the fermentation process to obtain new products with improved functional properties. In this frame, Pihurov et al. [7] tested the functionality of a co-culture of SCOBY-based membranes (Kombucha culture known as SCOBY-Symbiotic Culture of Bacteria and Yeasts) and milk kefir grains to produce functional bioactive compounds by fermenting a newly formulated black tea infusion medium supplemented with bovine colostrum and sugar. The chemical composition of the fermented product obtained under the optimized fermentation conditions positively impacted its functional properties in terms of antimicrobial and antioxidant properties. Indeed, the fermented product revealed high antibacterial activity against *Escherichia coli* and *Bacillus* spp., antifungal activity against *Aspergillus niger*, and a titratable acidity of 445 °Th (Thörner degrees).

Instead, Di Biase et al. [8] discussed the application of mathematical modeling to study and characterize *L. plantarum* ITM21B, a sourdough strain with pro-technological and functional features. This study aimed to determine the cardinal growth parameters for the strain’s pH, temperature, water activity, and undissociated lactic acid. Moreover, the strain growth, pH, total free amino acids, organic acids, and proteins were monitored under different fermenting conditions, e.g., microbial load, T, and pH, of a liquid sourdough based on wheat flour and gluten. The results showed that the strain growth and the metabolite pattern were affected by the fermentation conditions. The study highlighted that this mathematical predictive approach allowed for the simulation of the strain’s performance in different scenarios, being useful in optimizing the fermentation conditions needed to obtain the suitable nutritional and technological characteristics of *L. plantarum* ITM21B liquid sourdough.

Finally, food ontologies are becoming increasingly important in human nutrition research to standardize the terminology used and to create a machine-operable conceptual model. The study of Vitali et al. [9], using kefir and Parmigiano Reggiano as representatives of fresh and ripened dairy products, proposed such a semantic model for concepts related to consuming fermented foods from both a technological and health perspective to formalize the role of specific microbial taxa, and specific gene pathways, in the different steps of the dairy fermentation process. In this way, their model lays down a connection between the actors (taxa and pathways) involved in the raw ingredient transformation of fermented dairy products, connects them to resulting metabolites, and analyzes their consequences on the fermented product.

In conclusion, this Special Issue includes eight outstanding papers describing examples of the most recent advances in the applications of microbial processes and microorganisms themselves as alternative sources of nutritive and beneficial components for food products and derivatives thereof, exploring their bioactive component, as well as their biotechnological and techno-functional properties. The use of microorganisms and microbial processes in developing nutraceutical and functional foods has broad potential, and their applications can lead to novel, sustainable, and healthy food products. Further research in this field is essential not only to develop more effective and environmentally friendly approaches to apply with already studied microorganisms but also to identify new ones and to isolate microbial functional components.

## Data Availability

Data sharing not applicable.
